# Conductometric and Fluorescence Probe Analysis to Investigate the Interaction between Bioactive Peptide and Bile Salts: A Micellar State Study

**DOI:** 10.3390/molecules27217561

**Published:** 2022-11-04

**Authors:** Santosh Kumari, Suvarcha Chauhan, Ahmad Umar, Hassan Fouad, Mohammad Shaheer Akhtar

**Affiliations:** 1 Department of Chemistry, Himachal Pradesh University, Summer Hill, Shimla 171005, India; 2 Department of Chemistry, College of Science and Arts, Promising Centre for Sensors and Electronic Devices (PCSED), Najran University, Najran 11001, Saudi Arabia; 3 Department of Materials Science and Engineering, The Ohio State University, Columbus, OH 43210, USA; 4 Applied Medical Science Department, Community College, King Saud University, Riyadh 11433, Saudi Arabia; 5 School of Semiconductor and Chemical Engineering, Jeonbuk National University, Jeonju 54896, Korea; 6 Graduate School of Integrated Energy-AI, Jeonbuk National University, Jeonju 54896, Korea

**Keywords:** aggregation, bile salts, bioactive peptide, glycyl dipeptide, micellization

## Abstract

The present work deals with the micellar state study of sodium cholate and sodium deoxycholate in the aqueous solution of a bioactive peptide, namely glycyl dipeptide, having different concentrations through conductivity and fluorescence methods at different temperatures. The data obtained from conductivity is plotted against the concentration of Bile salts, and *CMC* (critical micelle concentration) values are calculated. The results realized have been elucidated with reference to Glycyl dipeptide–bile salts hydrophobic/hydrophilic interactions existing in solution. In addition, the *CMC* values converted to mole fraction $\left(X_{cmc}\right)$ values have been used to evaluate the standard thermodynamic factors of micellization viz., enthalpy H, free energy $\Delta G_{m}^{0}$, and entropy $\left(\Delta S_{m}^{0}\right)$ which extract information regarding thermodynamic feasibility of micellar state, energy alteration, and the assorted interactions established in the existing (bile salts–water–glycyl dipeptide) system. Furthermore, the pyrene fluorescence spectrum has also been utilized to study the change in micro polarity induced by the interactions of bile salts with glycyl dipeptide and the aggregation action of bile salts. The decrease in modification in the ratio of intensities of first and third peaks i.e., (I_1_/I_3_) for the pyrene molecules in aqueous bile salts solution by the addition of dipeptide, demonstrates that the micelle polarity is affected by glycyl dipeptide. This ratio has also been utilized to determine *CMC* values for the studied system, and the results have been found to be in good correlation with observations made in conductivity studies.

## 1. Introduction

Biologically significant macromolecules are the building blocks of living organisms and are prerequisites for all forms of life. As the research related to new chemical and pharmaceutical formulations has grown extensively in recent years, the industries are turning more progressively towards biologically active compounds such as peptides/proteins in search for better drug formulation [1]. In this regard, a considerable impetus is driven by the unique requirement of proteins/peptides, because they provide procedures that are more unwavering and have effectual bioavailability, and facilitate the sound development of drugs. Peptides are short polymers formed by the connection of (≤100) amino acids which encompass a few of the indispensable components of various biological processes of living beings, such as enzymes, antibodies, sundry hormones, etc. They are the significant building units that create the workhorses for living entities known as proteins [2,3]. The physiological action of a peptide is the manifestation of an assortment of interactions, which they endure with several metabolites present in the body of living beings. To facilitate discerning between these interactions, a logical acquaintance of solution behavior of such chemical constituents makes it possible to extend a more general idea of association and stability of bio-molecules such as amino acids, peptides, proteins, drugs, carbohydrates etc. in aqueous solutions [4,5]. It is therefore also a step closer to understanding protein self-aggregation, which perhaps is the reason behind several biological interactions.

**Figure 1:**
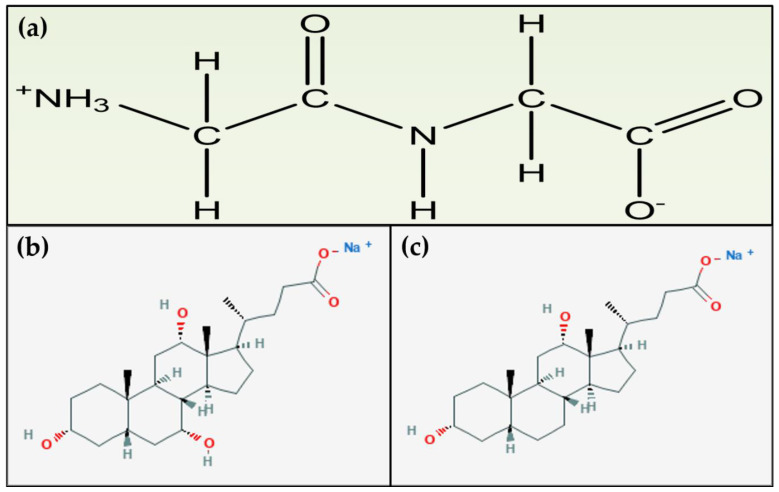
Molecular structure of (**a**) Zwitter ionic Glycylglycine, (**b**) sodium cholate (C_24_H_39_O_5_Na), and (**c**) sodium deoxycholate (C_24_H_39_O_4_Na).

**Scheme 1:**
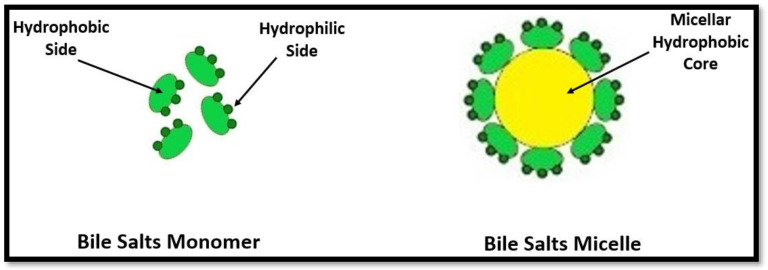
Schematic presentation of micelle formation.

Bile salts, the naturally occurring bio surfactants, are compounds containing steroid ring structures, which comprise the hydroxyl group (2/3) with the carboxylic group holding a side chain [6]. The steroidal framework of the molecules bear a puckered ring structure, where theCH_3_ groups (having a low affinity towards water) occupy the concave side, rendering the molecule amphiphilic characteristics, as shown in Figure 1. In aqueous solution at concentration near to their critical micelle concentration (*CMC*), the bile salts self-associate to make primary aggregates, which consists five to ten monomers, where the convex side encounters the internal part of the aggregate and the concave side encounters the aqueous part [7,8]. The schematic presentation of micelle formation is shown in Scheme 1. Furthermore, at a higher concentration these primary aggregates again assemble to produce secondary aggregates mediated through hydrophobic interactions and hydrogen bonding. They are naturally prevailing amphiphiles that are synthesized in the liver and play an imperative role in the solubilization of large hydrophobic constituents, such as fats in living organisms [9,10]. They have received attention for their inimitable characteristics, for instance their surface activity, self-aggregation, and solubilization of aquaphobic molecules [11], and have many uses in the petrochemical, food, cosmetic, agrochemical, pharmaceutical, paint, textile, and coating industries, being used as emulsifiers, solubilizers, suspension stabilizers, and wetting and foaming agents [12,13,14,15]. Thus, the possibility of enhancing the use of the surfactants in various fields has motivated much contemporary research.

Furthermore, in contradiction to conventional surfactants, bio surfactants (bile salts) possess a particular structure and stiff steroid backbone with an extended alkyl chain of numerous lengths ending with the carboxylic acid group, which leads to a distinctive aggregation behavior of bile salts and features which are more advantageous than conventional surfactants [16,17]. They are small aggregates which help to solubilize and disperse hydrolyzed fat and lipids that are derived from food. Bio-surfactants have a lesser amount of toxicity and are formed from renewable substrates, and many display stabilities under some conditions, viz. pH, temperatures, and ionic strength [18,19]. Interestingly, studies on micellar aggregates of bile salts help us to understand the interactions of biological membranes, hydrolysis, biliary secretion, and solubilization of cholesterol and hence are able to disclose certain physiological processes [20,21,22]. In this paper, we intend to investigate the micellar action of bile salts, viz., sodium cholate (NaC) (C_24_H_39_O_5_Na) and sodium deoxycholate (NaDC) (C_24_H_39_O_4_Na), in aqueous glycyl dipeptide, for attaining the proper understanding of their properties and applications in various fields viz. chemical, biochemical, pharmaceutical and industrial fields. The system has been analyzed in terms of variation in the micellar behavior of NaC and NaDC in the presence of glycyl dipeptide, by investigating critical micelle concentration (*CMC*) and related parameters. The analyzed system consists glycyl dipeptide, which may be considered to be the model component of enzyme and may further give valuable information on the interactive nature of biologically important compounds consisting of protein-bile salt. The study may further lend a hand in understanding the self-aggregation mechanism of bile salts in the presence of proteins/enzymes, which may be helpful in explaining the several biological interactions and processes. The molecular structures of these compounds are shown in Figure 1. Several physicochemical properties, for instance *CMC*, aggregation number, degree of dissociation, thermodynamic parameters, and micellar properties of bile salts, etc., have been frequently analyzed with the support of various investigating approaches, for instance, conductivity, density, fluorescence, surface tension, viscosity, diffusion, light-scattering, electron (nuclear) spin resonance methods (E(N)SR), osmometry, and refractive index measurements [23,24,25,26]. Moreover, the micelle formation of bile salts is affected by their external conditions, including the presence of co-solutes [27,28,29,30,31]. Thus, it is imperative to study the micellization properties of these surfactants that accommodate to manage the potency of various interactions affecting protein-surfactant systems.

## 2. Experimental Details

### 2.1. Chemicals

Distilled solvent water, used for the entire experiment, was obtained from a Millipore–Elix system with conductivity (2 to 3) µS∙cm^−1^ and pH~6.8−7.0 at temperature 298.15 K. Analytical grade NaC and NaDC were purchased from Himedia Pvt. Ltd. (Mumbai, India) and recrystallized from ethanol as per the method conveyed in our previous studies [29].The glycyl dipeptide was obtained from Spectrochem Pvt. Ltd. (Mumbai, India) and was utilized by itself without applying any supplementary action. Pyrene of A.R. grade, used as a fluorescent probe, was obtained from Merck (Darmstadt, Germany) and utilized without passing through any treatment. Molecular weight, purity, and sources of the chemicals used in our study are provided in Table 1.

### 2.2. Experimental Process

#### 2.2.1. Conductivity Measurements

The desired solutions of sodium cholate (NaC) (1–20 mmol∙kg^−1^) and sodium deoxycholate (NaDC) (1–10 mmol∙kg^−1^) were made in aqueous stock mixture of Glycyl dipeptide (0.001, 0.005, and 0.010 mol∙kg^−1^). The chemicals were weighed using a Shimadzu scalewith the precision of ±0.0001 g. Conductivity was measured via digital conductivity meter Cyberscan CON 510, whose procedure and principle of working has been elucidated earlier [32]. The temperature was upheld constantly at ±0.1 K by flowing thermostated water through a double-walled conductivity vessel containing the solution. The reproductivity of the conductivity measurements was assessed to be ±15 µS∙cm^−1^.

#### 2.2.2. Fluorescence Measurements

The fluorescence spectral analysis was completed with an LS–55 Perkins Elmer Fluorescence Spectrophotometer. The principle of working and procedure isdescribed in our prior study [31]. The samples were analyzed by using a quartz cuvette with a 10 mm path length. The wavelength for excitation was set aside at 334 nm and for the emission at 373 and 384 nm. The excitation slit was managed at 8.0 nm while the emission slit was kept at 2.5 nm. The pyrene solutions (2 µmol kg^−1^) utilized as a probe were prepared by following the procedure documented in the literature [31].

## 3. Results and Discussion

### 3.1. Conductivity Studies

#### 3.1.1. Micellization of Bile Salts in Aqueous Medium of Glycyl Dipeptide

**Figure 2:**
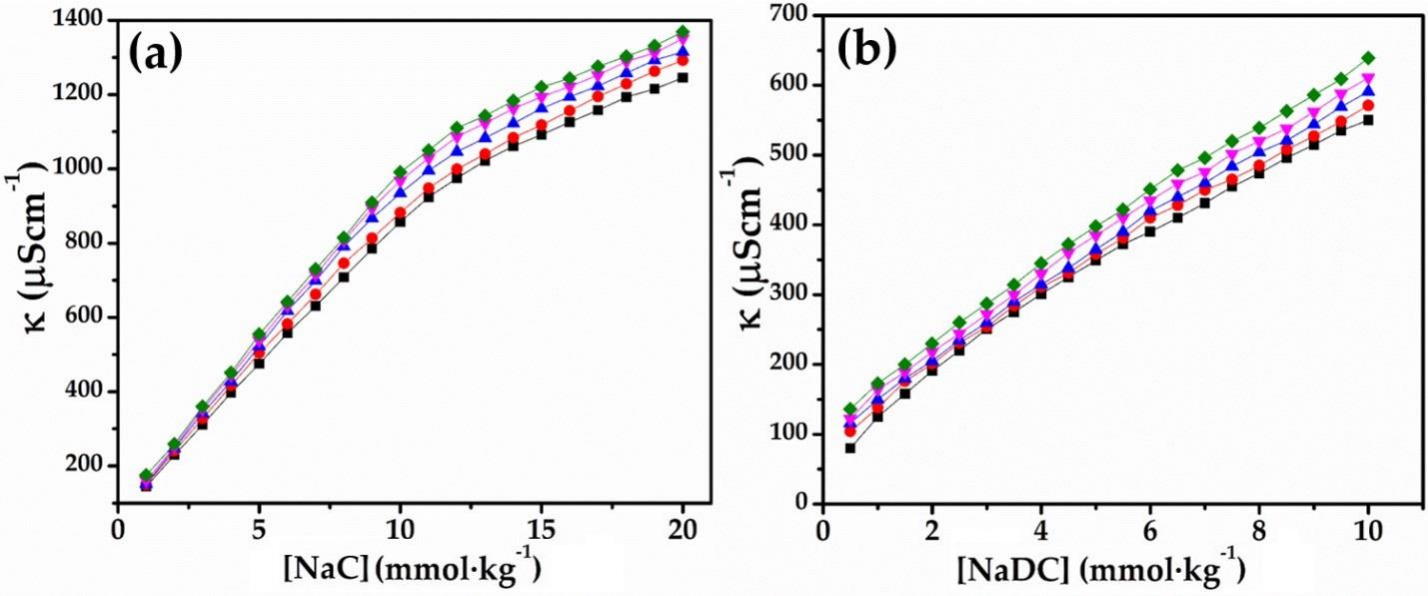
Illustrative plots of *κ*, as a function of concentration of (**a**) NaC and (**b**) NaDC in aqueous 0.010 mol∙kg^−1^ Glycyl dipeptide at 298.15 K (■), 303.15 K (●), 308.15 K (▲), 313.15 K (▼), and 318.15 K (◄).

This section employs the conductivity method to analyze the effect of micellization on the interaction of NaC and NaDC with glycyl dipeptide in an aqueous medium at fluctuating amounts (0.001, 0.005 and 0.010 mol∙kg^−1^) and temperatures (from 293.15 K up to 313.15 K). The conductivity data of NaC and NaDC in the aqueous solution of glycyl dipeptide at different temperatures are encapsulated in Table S1 in the supporting data. The illustrative plots between *κ* [NaC/NaDC] are presented in Figure 2. The plot displays a kink at the point of aggregation i.e., critical micelle concentration (*CMC*). The (*CMC*) values of NaC as well as NaDC have been extracted and are then turned into their mole fraction unit, *X_cmc_*, before exposing these values for a discussion of energetic micelle formation. However, it is important to mention that the *κ* values increase linearly as a function of bile salt content, prior (i.e., pre-micellar) and subsequently (i.e., post-micellar) to the point of aggregation, i.e., critical micelle concentration (*CMC*), for all studied temperatures, as shown in Figure 2. However, the *CMC* in the conductivity curves for NaC and NaDC cannot be identified clearly as the change in conductivity near the *CMC* region is not very fine, which has a characteristic quality when coupled with bile salts [33,34] and with some binary mixtures of conventional ionic surfactants. The elevation in *κ* values occurs more in pre-micellar than post-micellar regions, because the conductivity of bile salts solution results from the dependence of conductivity upon ions present on surfactants’ heads as well as the mobility of ions. At lower concentrations, NaC and NaDC act as strong electrolytes and undergo complete dissociation into ions and are free to move, therefore contributing significantly toward conductivity value. Furthermore, as the bile salts content increases, the association of bile salt initiates leads to the construction of self-organized molecular assemblies called micelles [35]. The aggregation and incorporation of counter-ions into these aggregates cause a diminishment in the number of free ions and hence a smaller increment in conductivity values [36,37]. However, the conductivity *κ* values are of higher magnitude for sodium cholate (NaC) in contrast to sodium deoxycholate (NaDC) for all the experimented systems. This can be explained by considering the structural relationship between NaC and NaDC, as NaDC has one less –OH group and hence lesser number of ions will be present for the movement, resulting in lower conductivity values. Moreover, the conductivity values escalate with the concentration of glycyl dipeptide for both the bile salts, and have been found to increase with bile salt concentration. The increase in conductivity values with bile salt concentration may be attributed to the fact that the number of charge carriers (ions) increases with addition of bile salts as well as glycyl dipeptide [37].

#### 3.1.2. Critical Micelle Concentration (*CMC*)

Furthermore, on the investigation of *CMC* values from Table 2, we infer that values for bile salts in pure water are similar to those in the literature [38,39,40,41], decreasing while proceeding towards higher concentrations of glycyl dipeptide. This leads to the following outcomes on the addition of glycyl dipeptide:

1. There is a decrease in the thickness of the solvation layer surrounding the (ionic) head groups of the bile salts.
2. The electrostatic repulsive kind of interactions are also lessened amongst the negatively charged part of the bile salts.

These two outcomes are responsible for the net diminution in the hydrophilicity of NaC and NaDC, i.e., their adsorption on the surface is increased and molecules assemble effortlessly on the surface and within the solution and hence *CMC* values decrease. Herein, it is imperative to remark that the dipeptides typically occur in the zwitterion and diffuse into ions alike amino acids, for the reason that dipeptide bonds do not detach. This may firmly lead towards the formation of ion pairs among differently-charged head assemblies of bile salts and molecules of glycyl dipeptide, by means of electrostatic interactions triggering solubilization of the glycyl dipeptide. Nevertheless, it has been noticed that the solubilization for the ion-pairs interaction of the glycyl dipeptide is privileged for NaC compared to NaDC; this can be attributed to the lack of –OH group in the latter, which makes it more hydrophobic. Precisely, glycyl dipeptide has been known to play a dual role, as it also reinforces the arranged solvent molecules surrounding the steroidal backbone of bile salt monomers with water molecules [38] or else the adding of glycyl dipeptide molecules lessens the electrostatic repulsive-type interactions among polar head parts of NaC/NaDC molecules and the ionic group of glycyl dipeptides. Moreover, the structured water surrounding of the steroidal backbone of bile salt monomers may have been ruptured withthe addition of glycyl dipeptide molecules [39]. This fortifies the solvophobicity of the studied bile salts system and establishes an easier micelle formation, thus, a steady decrease was observed for the *CMC* values. The uncertainty in the *CMC* values was ±0.03 × 10^−3^ and ±0.02 × 10^−3^ for NaC and NaDC, respectively.

#### 3.1.3. Temperature Dependence of *X_CMC_* (or *CMC*)

**Figure 3:**
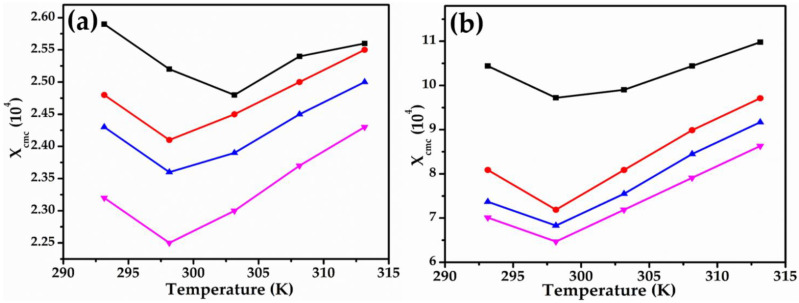
Illustrative plots of $X_{CMC}$ vs. temperature for (**a**) NaC and (**b**) NaDC in water (■), and 0.001 (●), 0.005 (▲) and 0.010 (▼) mol∙kg^−1^ aqueous glycyl dipeptide.

The variations of $X_{cmc}$ (or *CMC*) with temperature sheds light on micelle formation and micellar transitions to a large extent, and helps to identify the effect on the CMC of the amphiphiles. The $X_{cmc}$ values of bile salts in the considered solvent systems are recorded in Table 3 and displayed against temperatures in Figure 3. A careful scrutiny of these plots reveals that the values of $X_{cmc}\;$ confirm a deep minimum at around 298.15–308.15 K for NaC as well as for NaDC, and then escalate with rise in temperature. The variation in the $X_{cmc}$ values for NaC shows a typical U-type curve at the studied temperature range. However, for NaDC, a sheer decrease can be observed from Figure 3.The steepness in the $X_{cmc}$ curves for NaC and NaDC clearly expose that the micellization behavior of these bile salts is affected by temperature for all the concentrations. The larger diminishment in the $X_{cmc}$ values for NaDC along with temperature is the manifestation of the more hydrophobic character of NaDC. These types of characteristics for several ionic and non-ionic surfactants have already been suggested in the literature [40,41,42]. The temperature dependence on the values of $\;X_{cmc}\;$ for these bio-surfactants in aqueous systems can be construed as hydrophobic and likewise hydrophilic hydrations [43,44,45]. In the pre-micellar region, both the hydrations (i.e., hydrophobic along with hydrophilic hydrations) are equally important, whilst the hydrophilic hydrations are feasible in the post-micellar region of amphiphilic systems. After micellization, hydrophobic and hydrophilic hydrations decrease with temperature [43]. A lessening of hydrophilic hydration promotes the formation of aggregates (micelles), whereas the lessening of hydrophobic hydration demotes the self-assembling of amphiphilic molecules on escalating temperatures [45]. Subsequently, the supremacy of these two outcomes will be decisive factors for governing the magnitude of the *CMC* (or $\;X_{cmc}$) values in the studied temperature range. The uncertainty in the *X_CMC_* values was ±0.0002 × 10^−3^ and ±0.0001 × 10^−3^ for NaC and NaDC, respectively.

Alternatively, the increase in *CMC* values for studied bile salts in aqueous solutions of glycyl dipeptide could also be ascribed to the thermal motion of existing species in the ternary solution. It has also been observed that, with escalation in temperature, the kinetic energy of the molecules (bile salts and solvent) piles up, owing to the upsurge in the thermal motion of these molecules which ensures that the rupture of the water structure and the formation of micelles becomes intricated. Thus, with the elevation in temperature, disaggregation of micelles occurs; consequently, there is an increase in the $X_{CMC}$ or *CMC* values for the studied system. 

#### 3.1.4. Thermodynamics of Micellization of NaC and NaDC in Aqueous Glycyl Dipeptide

The distinctive thermodynamic parameters in relation to the micellization process of NaC and NaDC in analyzed solvent system have been calculated using the $X_{CMC}$ data summarized in Table 3. The standard enthalpy for the formation of micelles, $\Delta H_{m}^{0}$ for NaC and NaDC have been estimated by using the Equation (1) [46,47]:

(1) $$\Delta H_{m}^{0}=-RT^{2}\left(2-\alpha \right)[d\left(lnX_{cmc}\;\right)/dT$$

where $\;d(\ln X_{CMC}/dT)$ is the slope of *lnX_CMC_* versus *T* plots and α implies the degree of counter–ion dissociation, given as (2).

(2) $$\alpha =S_{2}/S_{1}$$

where *S*1 and *S*2 signifies the slopes before and after the *CMC* region, respectively, calculated by subjecting conductivity data to linear regression, and having a correlation factor greater than 0.998. The standard free energy of micellization, $(\Delta G_{m}^{0}$) nd entropy of micellization, $(\Delta S_{m}^{0}$, have been calculated from the following equations [46]:

(3) $$\Delta G_{m}^{0}=\left(2-\alpha \right)RTlnX_{cmc}$$

(4) $$\Delta S_{m}^{0}=\frac{\left(\Delta H_{m}^{0}-\Delta G_{m}^{0}\right)}{T}$$

**Figure 4:**
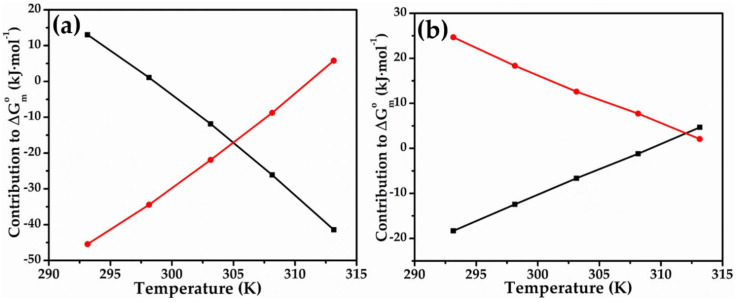
Illustrative plots of contribution of enthalpy, $\Delta H_{m}^{0}$ (■) and entropy, $-T\Delta S_{m}^{0}$ (●) to $\;\Delta G_{m}^{0}$ of (**a**) NaC and (**b**) NaDC in 0.10 mol∙kg^-1^ aqueous glycyl dipeptide.

The values of $\;\Delta H_{m}^{0}$, $\Delta G_{m}^{0}$, and $\Delta S_{m}^{0}$ for NaC and NaDC in different concentrations of glycyl dipeptide are tabulated in Table 4. In addition, their variations with temperature are shown in Figure 4.

**Table 4:** Counter ion dissociation, α and thermodynamic parameters, $\Delta H_{m}^{0}$ (kJ∙mol^−1^), $\Delta S_{m}^{0}$ (kJ∙K^−1^∙mol^−1^) and $\Delta G_{m}^{0}$ (kJ∙mol^−1^) for NaC and NaDC in studied concentration of aqueous glycyl dipeptide at various temperatures and experimental pressure, *p* = 0.1 MPa.

T/K	NaC				NaDC			
	** α **	**$\Delta H_{m}^{0}$** (kJ∙mol^−1^)	**$\Delta S_{m}^{0}$** (J∙K^−1^∙mol^−1^)	**$\Delta G_{m}^{0}$** (kJ∙mol^−1^)	** α **	**$\Delta H_{m}^{0}$** (kJ∙mol^−1^)	**$\Delta S_{m}^{0}$** (J∙K^−1^∙mol^−1^)	**$\Delta G_{m}^{0}$** (kJ∙mol^−1^)
Water								
293.15	0.804 (0.790) ^a^	6.72 (5.18) ^a^	0.105 (0.099) ^a^	−24.07 (−24.06) ^a^	0.677 (0.662) ^a^	31.32 (22.92) ^a^	0.207 (0.179) ^a^	−29.40 (−24.06) ^a^
298.15	0.803 (0.791) ^a^ (0.850) ^b^	3.17 (1.79) ^a^ (0.97) ^b^	0.093 (0.088) ^a^ (0.086) ^b^	−24.59 (−24.50) ^a^ (−24.60) ^b^	0.667 (0.668) ^a^ (0.780) ^b^	18.11 (15.74) ^a^ (0.57) ^b^	0.163 (0.154) ^a^ (0.098) ^b^	−30.41 (−30.13) ^a^ (−28.50) ^b^
303.15	0.799 (0.780) ^a^	−0.65 (−1.86) ^a^	0.081 (0.077) ^a^	−25.10 (−25.14) ^a^	0.762 (0.761) ^a^	03.44 (0.757) ^a^	0.107 (0.120) ^a^	−28.89 (−28.68) ^a^
308.15	0.847 (0.828) ^a^	−4.55 (−5.55) ^a^	0.065 (0.062) ^a^	−24.46 (24.29) ^a^	0.774 (0.765) ^a^	−10.76 (0.00) ^a^	0.059 (0.095) ^a^	−29.02 (−29.12) ^a^
313.15	0.861 (0.834) ^a^	−8.63 (−9.50) ^a^	0.051 (0.048) ^a^	−24.53 (−24.65) ^a^	0.756 (0.755) ^a^	−26.23 (−08.11) ^a^	0.012 (0.069) ^a^	−29.86 (−29.76) ^a^
[Glycyl Dipeptide] = 0.005 mol∙kg^−1^								
293.15	0.877	−6.13	0.135	−46.46	0.848	11.53	0.156	−34.13
298.15	0.919	−6.35	0.135	−47.17	0.821	1.40	0.122	−35.02
303.15	0.906	−6.56	0.134	−47.86	0.878	−9.47	0.086	−35.42
308.15	0.881	−6.77	0.133	−48.54	0.844	−21.00	0.048	−35.80
313.15	0.925	−6.98	0.132	−49.13	0.891	−33.75	0.010	−36.79
[Glycyl Dipeptide] = 0.005 mol∙kg^−1^								
293.15	0.907	−7.79	0.134	−47.92	0.734	14.39	0.168	−34.77
298.15	0.935	−8.05	0.133	−48.58	0.777	4.97	0.136	−35.58
303.15	0.939	−8.33	0.132	−49.28	0.796	−5.16	0.102	−36.07
308.15	0.945	−8.59	0.132	−49.91	0.721	−15.93	0.067	−36.49
313.15	0.92	−8.87	0.131	−50.58	0.803	−27.79	0.031	−37.50
[Glycyl Dipeptide] = 0.010 mol∙kg^−1^								
293.15	0.947	−11.37	0.123	−47.98	0.765	14.98	0.173	−35.72
298.15	0.952	−11.75	0.122	−48.60	0.765	6.11	0.143	−36.57
303.15	0.959	−12.18	0.12	−49.30	0.779	−3.40	0.110	−36.68
308.15	0.966	−12.6	0.119	−50.01	0.779	−13.49	0.077	−37.23
313.15	0.975	−12.95	0.118	−50.43	0.804	−24.55	0.044	−38.26

^a^ [38], ^b^ [48].

A fascinating feature about the data related to Table 4 is that the values of $\Delta H_{m}^{0}$ are endothermic (i.e., positive) at a lower temperature up to 303.15 K for both bile salts, and then turn to exothermic (i.e., negative) with a rise in temperatures for all the studied systems of glycyl dipeptide, irrespective of the variation in concentration. The larger lack of the –OH group in NaDC compared to NaC can be clearly noticed from more positive ∆$H_{m}^{0}$ values for NaDC for all the concentrations of glycyl dipeptide. The micellization process becomes more entropy-controlled for NaDC than that of NaC, as revealed by the fact that the transferring of NaDC molecules is associated with more breakages of the solvent structure to the micellar region, altogether explaining (for NaDC) > $\Delta S_{m}^{0}$ (for NaC). This seems to be steady with the actuality that the solubilization of glycyl dipeptide in NaDC is more than NaC, and thus, signifying larger involvement of glycyl dipeptide towards the thermodynamics process of micellization for NaDC. 

Figure 4 depicts that the $\Delta H_{m}^{0}$ and $\;\Delta S_{m}^{0}$ values in aqueous and various concentrations of glycyl dipeptidedecline with an upsurge in temperature. This behavior is vindicated by the fact that the hydrophobic dehydration (being accountable for more positive $\Delta S_{m}^{0}$ values at low temperatures) displays the role of hydrophobic type interactions in micellization. Similar results have been found for bile salts in binary solvent mixtures in aqueous systems [49]. This effect remains more effective for NaDC as attributed to larger positive $\Delta S_{m}^{0}$ values. Further, the decrease in $\Delta S_{m}^{0}$ values with the rise in temperatures can be manifested by the lessening of hydrogen bonding in spatial structures of water. Subsequently, when the temperature is elevated, less energy is needed in the disruption of spatial arrangements, owing to which more negative $\Delta H_{m}^{0}\;$ values being observed [49]. Both the bile salts seem to add their existence to this outcome, as recognized by more negative $\Delta H_{m}^{0}\;$ values in the presence and absence of glycyl dipeptide (Table 4). Similar outcomes have been revealed by others [50] which suggested that the dispersion forces of interactions responsible for negative $\Delta H_{m\;}^{0}$ values, which characterize the foremost attractive force in the process of micellization.

The $\Delta S_{m}^{0}$ values become positive under the examined experimental temperature, which makes the process of micellization for bile salts entropically favorable. As we know, micellization is a process where the system endures transformation from a monomeric state to micellar state, and thus a decline in $\Delta S_{m}^{0}$ values have been observed. However, more positive $\Delta S_{m}^{0}$ values have been observed due to breakage in the arranged structure of solvents around the hydrophobic chains, leading to more randomness in the mixture [51]. However, the decline in $\Delta S_{m}^{0}$ values with temperature may be explained in terms of the enhanced randomness of hydrophobic chains in mixture solution as a result of breakage of the ordered structure of solvent molecules [48].

In view of the evidence reported above, it is definite that, during the micelle formation of bile salts, entropy driven at lower temperatures and enthalpy controlled at higher temperatures occurs in different investigated concentrations of glycyl dipeptide. It was also observed that hydrophobic as well as electrostatic interactions between bile salts and glycyl dipeptide seem to be the outcomes of the temperature effect on $\Delta H_{m}^{0}\;$ and $\Delta S_{m}^{0}$ values. A similar effect has also been reported in the literature [52,53] for $\Delta H_{m}^{0}$ and $\Delta S_{m}^{0}$ values for ionic surfactants 

It can be inferred from Table 4 that $\Delta G_{m}^{0}$, values seem to be negative and lie within the range of −23 to −42 kJ∙mol^−1^ for diverse surfactants other those non-ionic in nature [53]. This displays the feasibility for spontaneous process at all the studied temperature ranges for the formation of micelles of NaC and NaDC in the studied system. The negative values of $\Delta G_{m}^{0}$ do not virtually depend on temperature orthe nature of glycyl dipeptide. This behavior is accountable for the compensation among $\Delta S_{m}^{0}$ and $\Delta H_{m}^{0}$ values and parting $\;\Delta G_{m}^{0}$ values nearly unaffected. $\Delta G_{m}^{0}$ is the summation of the ($\Delta H_{m}^{0}$) and ($-T\Delta S_{m}^{0}$) contributions, but as the temperature escalates, the enthalpy influence to the free energy rises, while the entropic influence declines, as revealed in Figure 4. Thus, the supremacy of entropy is shifted to enthalpy at the mid-temperature range analyzed for both bile salts. 

The above discussions on thermodynamic parameters have given an indication of a significant contribution towards the micellization process by the size of the hydrophobic assemblies. Thus, it is possible that the typical characteristic behaviors of glycyl dipeptide in the presence of NaC and NaDC has been observed, as a result of the hydrophobicity in the −R group as well as the hydrophilicity as a consequence of –NH_3_^+^ and –COO^−^ groups present in the glycyl dipeptide. This may be the prime aspect in estimating the energetic micelle formation of studied bile salts in aqueous glycyl dipeptide.

#### 3.1.5. Enthalpy–Entropy Compensation for Micelle Formation

Enthalpy–entropy recompense stretches linear reliance amidst the variation in enthalpy ($\Delta H_{m}^{0}$) and variation in entropy ($\Delta S_{m}^{0}$) and acts as a basic foundation for the thermodynamic investigation of the scarce and associated properties of micellization in numerous solvent systems [29,54]. The process of micellization was studied as an influence of following two consequences: (a) the “de-solvation” part, i.e., the dehydration of the hydrocarbon tail of surfactant molecules, and (b) the “chemical” part, i.e., the accretion of the hydrocarbon tails of surfactant molecules in the process of micelle formation [55].

Basically, the recompense phenomenon amid $\Delta H_{m}^{0}$ and $\Delta S_{m}^{0}$ in the numerous procedures is as follows [56]:

(5) $$\Delta H_{m}^{0}=\Delta H_{m}^{*}+T_{c\;}\Delta S_{m}^{0}$$

where $\;T_{c\;}$ is the compensation temperature and signifies the slope of the $\Delta H_{m}^{0}$ versus $\Delta S_{m}^{0}$ curve, and can be construed as solute–solvent interactions, i.e., anticipated as a degree of the “de-solvation” part in the process of self-aggregation. The intercept $\Delta H_{m}^{0}$ symbolizes the solute–solute interaction and is considered as per an index of the “chemical” part during micelle formation

**Figure 5:**
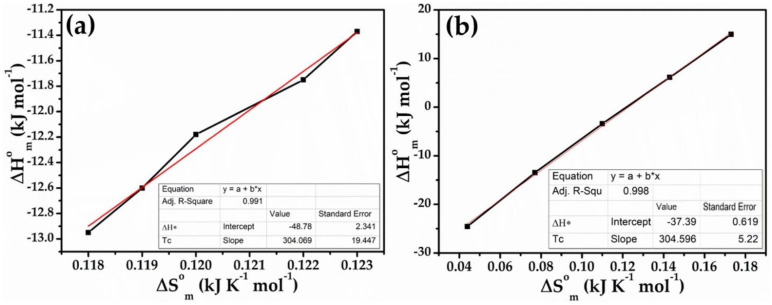
Descriptive plots for enthalpy-entropy compensation of (**a**) NaC and (**b**) NaDC in 0.010 mol∙kg^−1^ aqueous glycyl dipeptide.

In the present study, there seems to be an excellent relationship between the $\Delta H_{m}^{0}$ and $\Delta S_{m}^{0}$ values for NaC and NaDC at all the concentrations, with the correlation constantly falling within the range of 0.997 to 0.999, as revealed by Figure 5. The plots divulge that the compensation temperature $T_{c\;}$ (277.15–303.15 K) lies in a close conformity with the values given in the literature (270.15–300.15 K) [57], which recognize solvent structural deviations accompanying the process of micelle formation etc. [57,58,59]. The intercept $\Delta H_{m}^{*}$ decreased to some extent as we moved from values in water to aqueous glycyl dipeptide was at a smaller proportion in aqueous NaDC than the NaC system, i.e.,$\;\Delta H_{m}^{*}$ values are more negative for NaDC than NaC at all studied solvent systems. The factor $\;\Delta H_{m}^{*}$ is the enthalpy at $\Delta S_{m}^{0}$ = zero and points to the steadiness of micelles, thus, the higher the value of $\;\Delta H_{m}^{*}$, the lesser the firmness of the micelles [57]. Hence, the obtained results specify the involvement of chemical parts in micellization, and the stability of the micelle formed is improved with the addition of glycyl dipeptides, and this is more distinct with NaDC than NaC. Analogous enthalpy—entropy recompense has also been reported in the case of SDS—amino acid systems [60].

### 3.2. Fluorescence Probe Studies of NaC and NaDC

In this section, we explain fluorescence spectra for bile salts viz. NaC and NaDC for the estimation of the *CMC* values in aqueous and aqueous solutions of glycyl dipeptide at room temperature.

Pyrene, being a colorless solid used for the spectral measurements, is comprised of four fused rings (benzene), which results in a flat aromatic arrangement. Pyrene is prepared in partial organic complexes. The fluorescence spectrum of pyrene is quite sensitive to the polarity of the solvent system, so it is used as a probe to assess the environments of the solvent. This is because its excited state has non-planar shape, unlike that of the ground state. Definite emission bands remain unaffected, but others may vary in intensity owing to the strength of various interactions with the solvent.

**Figure 6:**
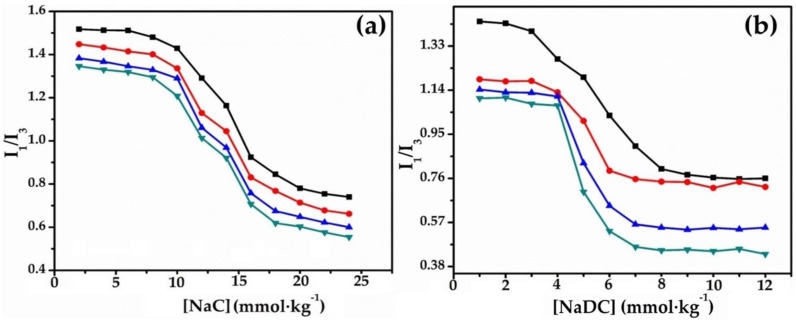
Plots of I_1_/I_3_ versus concentration of (**a**) NaC and (**b**) NaDC in pure water and in 0.001 (■), 0.005 (●), and 0.010 (▲) mol∙kg^-1^ glycyl dipeptide.

The I_1_/I_3_ dependence of pyrene on the content of NaC and NaDC is demonstrated in Figure 6. The I_1_/I_3_ remains unchanged up to a definite concentration of bile salts and then drops down abruptly above it. The first break points in the fluorescence spectra investigation by using pyrene solution as a probe are due to the solvent reliance of the vibrational band intensities of the pyrene fluorescence spectra. The fluorescence spectra of pyrene display five emission peaks at 373, 379, 383, 389, and 393 nm, detected for pyrene solution (2 µmol·kg^−1^) and fluorescence spectra in water [61]. It has been perceived for pyrene that the fraction of intensity of I_1_ to I_3_ at 373 and 384 nm, respectively, is a sensitive parameter to characterize the polarity around the probe’s environment. The lower value of the I_1_/I_3_ point to a polar environment [62,63,64] since pyrene has a lesser solubility in aqueous mediums (~10^−7^ mol·kg^−1^) than that of hydrophobic solvent (0.075 mol·kg^−1^). It is powerfully dispersed into micelles as quickly as they are formed, and since the conversion can be observed by a rapid decline in the ratio of I_1_/I_3_, we can assume the commencement of the micelle formation when the bile salts are mixed in the pyrene solution.

The plots of I_1_/I_3_ versus the amount of NaC and NaDC in water and aqueous glycyl dipeptide are illustrated in Figure 6. When the micelles are not formed, i.e., below *CMC* pyrene undergoes the polarity from the environment around the water molecules that ultimately result in higher values of the I_1_/I_3_ ratio. However, when the micelles are formed, i.e., above the *CMC*, pyrene molecules are solubilized into the interior part of the micelle due to their more hydrophobic nature. Thus, the environment around the pyrene senses a hydrophobic solvent, which has smaller polarity owning to a decrease of I_1_/I_3_ values. It has been observed from the plots of I_1_/I_3_ that the values follow the sequence water > NaC > NaDC, which reflects the increasing nature of the hydrophobicity in the solvent system.

The values for *CMC* have been computed by plotting the ratio of I_1_/I_3_ against the concentration of bile salts by using the sigmoidal Boltzman equation (SBE) [64], where all the plots display sigmoidal nature. The *CMC* values for NaC and NaDC at lab temperature for the considered solvent systems are presented in Table 5. The *CMC* values measured by this method were also in a similar fashion to those found by conductance measurements. The results corroborate the observations obtained from the conductance measurements.

**Table 5:** *CMC* of NaC and NaDC in studied concentration of aqueous glycyl dipeptide at room temperature and experimental pressure, *p* = 0.1 MPa.

^a^ m/mol∙kg^−1^	*CMC*/mmol∙kg^−1^			
	Fluorescence Probe Study		Conductivity Study	
	NaC	NaDC	NaC	NaDC
water	14.1	5.3	14	5.4
0.001	13.5	4.1	13.3	4
0.005	12.9	3.9	13.1	3.8
0.010	12.6	3.8	12.5	3.6

^a^ m is the molality of Glycyl dipeptide in water.

## 4. Conclusions

The conductivity studies lead to a clear picture of the thermodynamic behavior of dipeptides; eventually the polypeptide and hence protein interactions in aqueous environment will also be estimated easily. As shown in the present case, the interaction strength of dipeptide with bio-surfactants increases with concentration increments but decreases with temperature, showing a strong dependence on these parameters. However, NaDC appears to decrease the *CMC* values to larger extent compared to NaC, due to its more hydrophobic nature and hence promotion of micellization. Furthermore, the results obtained from fluorescence spectroscopic studies corroborate with results obtained from conductivity studies. The aforementioned studies on Glycylglycine and NaC/NaDC thus seem to reveal the underlying facts about the micellar structures and the role played by them in physiological systems.

## Figures and Tables

**Table 1:** Various specifications of chemicals taken in the present study.

Chemical Name	Source	Mol.Wt./kg∙mol^−1^	Purification Method	Mass Fraction Purity ^a^
Glycylglycine (C_4_H_8_N_2_O_3_)	Spectrochem Pvt. Ltd.	0.132	None	0.98
Sodium cholate (C_24_H_39_O_5_Na)	Himedia Pvt. Ltd.	0.431	Recrystallization	0.98
Sodium deoxycholate (C_24_H_39_O_4_Na)	Himedia Pvt. Ltd.	0.415	Recrystallization	0.98
Pyrene (C_16_H_10_)	Merck	0.202	None	0.96

^a^ Declared by the supplier.

**Table 2:** *CMC* values of bile salts in studied concentration of aqueous glycyl dipeptide at various temperatures and experimental pressure, *p* = 0.1 MPa.

T (K)	*CMC*, 10^3^							
	NaC, mmol∙kg^−1^				NaDC, mmol∙kg^−1^			
	Water	0.001	0.005	0.010	Water	0.001	0.005	0.010
293.15	14.4 (14.4) ^a^	13.9	13.5	12.9	5.1	4.5	4.1	3.9
298.15	14.0 (14.1) ^a^ (13.8) ^b^ (12.9) ^c^ (16.0) ^d^	13.3	13.1	12.5	5.4	4.0	3.8	3.6
303.15	13.9 (14.0) ^a^	13.5	13.3	12.8	5.7	4.5	4.2	4.0
308.15	14.1 (14.1) ^a^	13.8	13.6	13.2	5.9	5.0	4.7	4.4
313.15	14.2 (14.3) ^a^	14.1	13.9	13.5	6.1	5.4	5.1	4.8

^a^ [38], ^b^ [39], ^c^ [40], ^d^ [41].

**Table 3:** *X_CMC_* values of bile salts in studied concentration of aqueous glycyl dipeptide at various temperatures and experimental pressures, *p* = 0.1 MPa.

T (K)	*X_CMC_*, 10^4^							
	NaC, mmol∙kg^−1^				NaDC, mmol∙kg^−1^			
	Water	0.001	0.005	0.010	Water	0.001	0.005	0.010
293.15	2.59	2.48	2.43	2.32	10.44	8.09	7.37	7.01
298.15	2.52	2.41	2.36	2.25	9.72	7.19	6.83	6.47
303.15	2.48	2.45	2.39	2.3	9.9	8.09	7.55	7.19
308.15	2.54	2.5	2.45	2.37	10.44	8.99	8.45	7.91
313.15	2.56	2.55	2.5	2.43	10.98	9.71	9.17	8.63

## Data Availability

Not applicable.

## Supplementary Materials

The following are available online at https://www.mdpi.com/article/10.3390/molecules27217561/s1, Table S1: Specific Conductance, *κ* (*µ* Scm^−1^) values for NaC and NaDC (mmol∙kg^−1^)in pure water and in 0.001, 0.05 and 0.010 mol∙kg^−1^ aqueous solution of glycyl dipeptide at different temperatures.
