# Supplementary material for: Conductometric and Fluorescence Probe Analysis to Investigate the Interaction between Bioactive Peptide and Bile Salts: A Micellar State Study
Source: Molecules. 2022 Nov 4;27(21):7561. doi: 10.3390/molecules27217561 (PMC9654833; doi:10.3390/molecules27217561)
Supplement: Supplementary file 1 [file molecules-27-07561-s001.zip › molecules-2009093-supplementary.pdf]

Supplementary Material

Conductometric and Fluorescence Probe Analysis to Investigate the Interaction between Bioactive Peptide and Bile salts: A Micellar State Study

Santosh Kumari [1], Suvarcha Chauhan [1,*], Ahmad Umar [2,3,*,†], Hassan Fouad [4] and Mohammad Shaheer Akhtar [5,6]

[1] Department of Chemistry, Himachal Pradesh University, Summer Hill, Shimla171005, India
[2] Department of Chemistry, College of Science and Arts, Promising Centre for Sensors and Electronic Devices (PCSED), Najran University, Najran11001, Saudi Arabia
[3] Department of Materials Science and Engineering, The Ohio State University, Columbus, OH 43210, USA
[4] Applied Medical Science Department, Community College, King Saud University, Riyadh11433, Saudi Arabia
[5] School of Semiconductor and Chemical Engineering, Jeonbuk National University, Jeonju 54896, Korea
[6] Graduate School of Integrated Energy-AI, Jeonbuk National University, Jeonju 54896, Korea
* Correspondence: scschauhan19@gmail.com (S.C.); ahmadumar786@gmail.com (A.U.)
† Adjunct Professor at the Department of Materials Science and Engineering, The Ohio State University, Columbus, OH 43210, USA.

Table S1. Specific Conductance, $\kappa(\mu Scm^{-1})$ values for NaC and NaDC(mmol·kg^{-1})in pure water and in 0.001, 0.05 and 0.010 mol·kg^{-1} aqueous solution of glycyl dipeptide at different temperatures.

NaC						NaDC					
**[NaC] / T**	**293.15 K**	**298.15 K**	**303.15 K**	**308.15 K**	**313.15 K**	**[NaDC] / T**	**293.15 K**	**298.15 K**	**303.15 K**	**308.15 K**	**313.15 K**
**[Pure Water]**											
**1**	249	264	232	255	256	**0.5**	35	40	35	35	40
**2**	308	319	297	320	322	**1.0**	65	70	65	65	70
**3**	371	370	354	381	373	**1.5**	95	100	95	100	100
**4**	419	429	428	438	448	**2.0**	125	130	124	128	130
**5**	477	481	483	499	508	**2.5**	155	160	153	160	160
**6**	538	537	546	558	590	**3.0**	182	191	185	190	190
**7**	596	593	611	618	648	**3.5**	213	218	220	220	220
**8**	652	653	677	689	712	**4.0**	243	248	250	245	250
**9**	703	708	725	750	777	**4.5**	275	274	280	280	280
**10**	767	762	766	801	830	**5.0**	305	305	310	305	310
**11**	835	827	823	863	890	**5.5**	335	335	335	335	340
**12**	882	875	869	911	935	**6.0**	360	358	365	365	370
**13**	927	916	912	965	985	**6.5**	385	380	385	385	400
**14**	967	947	959	1005	1027	**7.0**	405	405	410	405	425
**15**	999	978	1009	1049	1068	**7.5**	425	430	435	425	450
**16**	1032	1015	1043	1093	1112	**8.0**	445	455	455	450	475
**17**	1068	1053	1089	1139	1159	**8.5**	465	480	480	475	500
**18**	1098	1091	1135	1183	1210	**9.0**	485	499	500	499	525
**19**	1132	1130	1179	1233	1266	**9.5**	510	520	525	519	550

**20**	1176	1167	1232	1280	1323	**10.0**	530	540	550	539	575
**[Glycyl dipeptide] = 0.001 mol·kg^{-1}**											
**1**	106	109	122	131	141	**0.5**	38	49	54	61	69
**2**	180	200	214	224	236	**1.0**	71	77	84	92	99
**3**	245	265	285	299	322	**1.5**	98	106	115	121	131
**4**	306	322	347	368	402	**2.0**	131	139	148	157	169
**5**	366	390	420	441	472	**2.5**	161	168	178	188	199
**6**	415	445	485	506	549	**3.0**	187	194	209	219	231
**7**	474	510	558	589	625	**3.5**	219	223	239	255	269
**8**	532	590	625	661	699	**4.0**	247	252	270	289	305
**9**	596	640	690	732	778	**4.5**	277	286	300	319	339
**10**	657	726	751	799	836	**5.0**	308	322	335	349	366
**11**	725	788	819	868	898	**5.5**	338	351	365	375	390
**12**	780	840	885	922	951	**6.0**	361	375	392	401	413
**13**	825	899	930	968	988	**6.5**	388	395	413	422	435
**14**	871	915	965	1005	1032	**7.0**	408	416	435	447	461
**15**	909	961	1000	1052	1071	**7.5**	427	439	461	476	485
**16**	951	1003	1040	1090	1105	**8.0**	448	458	485	496	505
**17**	994	1042	1085	1120	1147	**8.5**	471	479	505	512	530
**18**	1040	1088	1127	1161	1182	**9.0**	489	496	530	541	555
**19**	1090	1137	1167	1202	1215	**9.5**	512	520	555	566	580
**20**	1125	1161	1200	1230	1261	**10.0**	535	542	580	599	609
**[Glycyl dipeptide] = 0.005 mol·kg^{-1}**											
**1**	129	133	142	149	158	**0.5**	59	75	93	105	111
**2**	212	221	232	243	252	**1.0**	88	98	121	132	142
**3**	285	301	315	333	353	**1.5**	119	135	155	172	191
**4**	347	371	395	411	433	**2.0**	149	165	186	211	229

**5**	420	446	473	493	520	**2.5**	179	200	223	241	264
**6**	485	512	551	575	612	**3.0**	215	230	253	276	295
**7**	558	584	631	661	688	**3.5**	245	260	291	309	326
**8**	625	651	703	741	772	**4.0**	272	295	321	334	349
**9**	690	723	778	808	846	**4.5**	295	325	343	359	374
**10**	751	781	845	879	913	**5.0**	316	350	365	382	395
**11**	809	852	906	940	978	**5.5**	345	369	384	400	412
**12**	868	911	959	990	1031	**6.0**	371	387	399	420	435
**13**	918	955	996	1030	1069	**6.5**	392	405	418	435	453
**14**	956	991	1041	1072	1113	**7.0**	417	425	434	454	469
**15**	996	1038	1082	1112	1148	**7.5**	438	445	457	473	489
**16**	1028	1075	1117	1149	1191	**8.0**	454	462	474	492	507
**17**	1065	1112	1154	1185	1226	**8.5**	476	485	499	514	526
**18**	1108	1147	1191	1221	1261	**9.0**	495	506	520	533	547
**19**	1152	1191	1226	1254	1301	**9.5**	521	525	539	554	568
**20**	1198	1226	1263	1289	1330	**10.0**	541	545	559	571	588
**[Glycyl dipeptide] = 0.010 mol·kg^{-1}**											
**1**	145	150	152	161	175	**0.5**	**80**	104	116	122	136
**2**	230	241	248	252	259	**1.0**	**125**	138	150	164	173
**3**	311	328	342	353	360	**1.5**	**158**	176	180	189	200
**4**	398	417	428	444	451	**2.0**	**191**	201	205	218	230
**5**	475	504	523	538	555	**2.5**	**220**	231	235	244	260
**6**	558	582	618	631	642	**3.0**	**251**	254	260	272	287
**7**	631	662	699	715	730	**3.5**	**275**	284	290	300	314
**8**	708	746	791	807	815	**4.0**	**301**	311	315	330	345
**9**	785	813	867	894	909	**4.5**	**325**	331	338	360	372
**10**	857	882	935	968	991	**5.0**	**349**	358	365	385	398

**11**	924	948	996	1029	1050	**5.5**	**372**	381	390	410	422
**12**	975	999	1046	1087	1110	**6.0**	**390**	410	420	435	451
**13**	1022	1040	1083	1123	1143	**6.5**	**410**	428	440	459	478
**14**	1061	1084	1122	1162	1183	**7.0**	**431**	450	460	476	496
**15**	1092	1118	1163	1194	1220	**7.5**	**455**	465	484	502	520
**16**	1126	1156	1194	1222	1244	**8.0**	**474**	485	504	520	539
**17**	1158	1195	1223	1252	1276	**8.5**	**496**	508	521	538	563
**18**	1193	1229	1258	1289	1303	**9.0**	**515**	527	544	562	586
**19**	1215	1263	1293	1312	1331	**9.5**	**535**	548	569	588	609
**20**	1245	1292	1315	1351	1369	**10.0**	**550**	571	591	611	639

Standard uncertainties, u, are u(T) = ±0.01 K, u(p) = ±0.002 MPa, u(m) = ±0.003 mol·kg^{-1}, u(κ) = ±2 μS·cm^{-1} and relative uncertainty in conductivity measurement i.e. $u_r(\kappa) = u(\kappa)/\kappa$ = 0.05 (level of confidence = 0.68).
